# Exploring Impact of Rare Variation in Systemic Lupus Erythematosus by a Genome Wide Imputation Approach

**DOI:** 10.3389/fimmu.2019.00258

**Published:** 2019-02-26

**Authors:** Manuel Martínez-Bueno, Marta E. Alarcón-Riquelme

**Affiliations:** ^1^Department of Medical Genomics, GENYO, Center for Genomics and Oncological Research Pfizer, University of Granada, Granada, Spain; ^2^Unit of Chronic Inflammation, Institute for Environmental Medicine, Karolinska Institute, Stockholm, Sweden

**Keywords:** SLE, systemic lupus erythematosus, imputated rare variation, GWAS—genome-wide association study, sequence kernel association test, aggregated case-control enrichment

## Abstract

The importance of low frequency and rare variation in complex disease genetics is difficult to estimate in patient populations. Genome-wide association studies are therefore, underpowered to detect rare variation. We have used a combined approach of genome-wide-based imputation with a highly stringent sequence kernel association (SKAT) test and a case-control burden test. We identified 98 candidate genes containing rare variation that in aggregate show association with SLE many of which have recognized immunological function, but also function and expression related to relevant tissues such as the joints, skin, blood or central nervous system. In addition we also find that there is a significant enrichment of genes annotated for disease-causing mutations in the OMIM database, suggesting that in complex diseases such as SLE, such mutations may be involved in subtle or combined phenotypes or could accelerate specific organ abnormalities found in the disease. We here provide an important resource of candidate genes for SLE.

## Introduction

Genome-wide association studies have been designed primarily to capture common variation and so far some 10,000 common genetic variants have been robustly associated with a wide range of complex diseases (1). Therefore, this methodology is underpowered to detect the effects of rare variants. There has been much debate as to the role of rare genetic variation on complex traits (2–4) and how rare variant studies complement GWASs (5). It is now accepted that rare variants located in different genes could in fact play a more important role in disease susceptibility than common variants (4). The challenge arises in measuring and statistically analyzing rare variation. It would be very unexpected to find rare variants that could have substantial effect sizes and therefore high penetrance contributing to complex traits, being more likely to have mutations with modest effects. For small effect sizes association testing may require composite tests of overall “mutational load,” pooling rare variants for analysis by addressing the question: do rare variants increase or decrease disease risk? (6). In-depth whole-genome sequencing is the most comprehensive approach for measuring rare and common variation in both coding and non-coding regions. However, nowadays, its application is limited by the costs and various computational challenges, especially for large-scale cohorts. Whole-exome sequencing is a cost-effective alternative, however one of its obvious drawback is the absence of variants in non-coding regions, which may be especially relevant in the context of complex disease genetics. Genotype imputation is likely to remain a valuable tool. At this point an interesting cost and a computationally effective alternative would be to combine genotype imputation with targeted sequencing in a gene-centered strategy. For dense genotyping arrays, imputation is able to predict nearly all missing common variation with high accuracy, but as the variant minor allele frequency (MAF) decreases, so does the accuracy of imputation, depending mainly on the size of the reference panel and the ancestry of the imputed samples, with the best efficiencies in European cohorts, mainly due to the sufficiently large size of the European reference panels (7–13).

We have previously tested the overall effect of imputed rare variation on particular genes in systemic autoimmunity (14). Recently, we have implemented and successfully applied a method based on genotype imputation of rare variation, on a set of genes detected by exome sequencing as possible candidates for association to systemic lupus erythematosus (SLE) by mutation in members of Icelandic SLE-multicase families (15).

In the present work the method has been brought to the genome level to scan for association of protein coding genes with SLE by rare variation in the European ancestry population. We executed stringent imputation in a densely genotyped set for analyzing association with SLE in a sample from the European population selecting the protein coding genes of the genome and then applying tests to detect those that significantly associated with the disease by rare-variation. This procedure provided a set of 98 genes as good candidates for association with SLE by mutation. Many of these genes showed immunological related functions or effects on other organs or tissues affected by the pathology, such as joints, skin, central nervous system or blood and some were involved in human energy metabolism and more specifically as part of the respiratory chain. Such a diversity of functions is to be expected because of the phenotypic heterogeneity of a complex disease as SLE.

## Materials and Methods

### Genome-Wide Association Analysis

We used GWAS data from 5,478 individuals of European ancestry including 4254 SLE patients and 1,224 controls genotyped using the Illumina HumanOmni1_Quad_v1-0_B chip. In order to increase the number of controls, additional data from European subjects were obtained from the dbGaP database (http://www.ncbi.nlm.nih.gov/gap) including the DCEG Imputation Reference Dataset (phs000396.v1.p1) with 1,175 individuals representing general population, and controls subjects from two case-control studies: 1,047 from the High Density SNP Association Analysis of Melanoma (phs000187.v1.p1) and 903 from GENIE UK-ROI Diabetic Nephropathy GWAS (phs000389.v1.p1). Note that only control from both case-control studies were included in our analysis. In total, the initial dataset consisted of 4,254 SLE patients and 4,349 controls.

In order to obtain a quality-controlled working dataset satisfying current state-of-the-art criteria for association studies, data filtering was conducted using PLINK v1.07 1 (16) applying the following criteria: minimum total call rate per sample of 90%, minimum call rate per marker of 98%, minor allele frequency (MAF) threshold of 1%, Hardy-Weinberg Equilibrium (HWE) *p*-value for cases and controls at a minimum of 0.0001, and in addition at 0.01 only for controls, and finally a cutoff *p*-value of 0.00001 for differential missingness in, the software REAP was used (17) applying a kinship coefficient threshold < 0.055. To correct for stratification, principal component analysis (PCA) was performed with smartpca, EIGENSOFT 4.0 beta package 2 (18). To confirm the European ancestry we ran a PCA with the set of independent markers (*r*^2^ < 0.1) that maximized differences in allelic frequencies between the four main 1000 Genomes subpopulations (EUR, AFR, AMR, and ASN). No samples were detected as “non-European” (Supplemental Figure 1). Next, the PCA was performed with the set of *r*^2^ < 0.1, that maximized differences in the allelic frequencies in 1000 Genomes EUR subpopulations (CEU, GBR, IBS, TSI, and FIN) and two additional subpopulations from our sample dataset, Greek and Turkish (GRK and TUR) representing the south-eastern European ancestry. The resulting PCs perfectly classified the individuals from reference populations by their geographical origin (Supplemental Figure 2). This last set of PCs was used for correcting for genetic stratification in the case-control association analysis (Supplemental Figure 3). This resulted in a λ_GC_ = 1.05 using the first 10 principal components. The final data set used for association analysis consisted of 4,212 cases and 4,065 controls.

### Imputation

Release 19 (GRCh37.p13) was used as reference (https://www.encodeproject.org/files/gencode.v19.annotation/). Of 19,430 sequences annotated as “protein coding” in the gencode.v19.annotation.gtf file, 15,763 included in the final QC-filtered genotyping data set became our imputation working gene list. Each protein-coding gene region was extended 500,000 additional base pairs upstream and downstream, respectively, as it is known that large buffers may improve accuracy for low-frequency variants during imputation. Markers within each extended region were extracted from the GWAS data for imputation with IMPUTE2 (19) using the 1000 Genomes Project as reference panel. Specifically, we used 1000 Genomes Phase 3, b37 (October 2014), as these haplotypes have lower genotype discordance and improved imputation performance into downstream GWAS samples, especially for low frequency variants (20, 21). Genome-Wide Association Analysis of Imputed Rare Variants in complex diseases has been previously used as a gene-centered approach (7). In this paper imputation into a GWAS scaffold using the WTCCC European analysis cohort explicitly showed substantial gains in power to detect rare variant association within the gene where the extent of the increase in power depends crucially on the number of individuals in the reference panel. Therefore, power gains obtained from 500 to 4,000 samples in the reference panel were not as great as from 120 to 500 samples. Based on this, and for the objective of our study we considered as adequate the 2,504 samples present in the 1000 Genomes phase 3 reference panel (2014 release, http://mathgen.stats.ox.ac.uk/impute/1000GP_Phase3/) of which 503 are of European descent. Prior to imputation, each GWAS gene extended region was phased with SHAPEIT using the 1000 Genomes EUR subpopulation as reference (http://www.shapeit.fr/). A restrictive QC-filter was applied on the imputed genotypes (SNP genotyping rate ≥ 99%, sample genotyping rate ≥ 95%) without restriction of allele frequencies, in order to include both rare and low frequency variants. To ensure a highly reliable imputation, a conservative IMPUTE info_value threshold of ≥ 0.75 for each marker were applied as imputation quality score.

### Functional Annotation of Genetic Variants

Annotation of analyzed genetic variants in their different functional categories was carried out using ANNOVAR (22).

### Gene Case-Control Association Analysis by Rare Variation

While there is no universally accepted definition of “rare variant,” and a minor allele frequency (MAF) of 1% is the conventional definition of polymorphism, then a MAF < 1% would be understood as “rare variation.” We tested whether any of the N genes in the human genome had statistical evidence of association with SLE in the general European population due to the combined effect of all rare variation within each gene (MAF < 1%). Each gene was analyzed using two procedures: the sequence kernel association (SKAT) test (20, 21) and a case-control burden test by adjusting a logistic regression model with a “transformed” “genetic variable” equals to the sum of minor frequency alleles for all markers below the (< 1%) in the tested gene in each *i* individual (7). To note that in such case-control burden test, a result statistically significant indicates that the overall effect of rare variation on the gene goes in the same direction being either of risk (aggregate odd ratio > 1) or alternatively protective (aggregate odd ratio < 1). This feature of case-control burden analysis helps to interpret the effect of rare variation on the phenotype. In addition, running two association procedures, SKAT ⋂ case-control burden test would reduce the rate of false positives. Thus, we will consider as true positives those genes with significant association test for both procedures, SKAT and case-control burden test. However, in association tests that simultaneously include several markers, one effect of linkage disequilibrium (LD) between these markers could be collinearity. We have addressed the LD issue running the tests with a set of independent markers by applying a very restrictive LD threshold of *r*^2^ < 0.1. It could be argued that association signals would be lost by applying such a strict threshold of *r*^2^ but even so, if the signal remains it supports it as “true positive” (15). Supplemental Figure 4 summarizes the study workflow.

### Correcting for Stratification in Rare Variant Association Analysis

We verified that the set of Principal Components computed with common variation was able to correct stratification for rare variant association analysis in our sample (15). To be as stringent as possible, the 10 first principal components (PC's) and genomic control (GC) were used to correct for stratification in both tests. For case-control burden 10 PC's corrected tests, the genomic inflation factor (λ_GC_) was equal to 1.11 and for SKAT 10 PC's corrected tests it was equal to 1.24. These λ_GC_ values were used for correction of the resulting inflation on each type of association test (GC correction = Statistic ${}_{10\text{P}{\text{C}}^{\text{′}}\text{c}\text{\_}\text{corrected}}$/ λ_GC_). When no PC's correction was used, the λ_GC_ for case-control burden tests was equal to 1.44 and 2.97 for SKAT. Thus, the 10 PC's correction reduced the inflation by 33% in the case-control burden tests, and in 174% in the SKAT tests.

### Correcting for Multiple Testing in Gene Case-Control Association Analysis of Rare Variation

Regarding the question of correcting for multiple testing in gene association by rare variants, a genomic association threshold of 10^6^ is accepted (equivalent to Bonferroni correction for 19,000–20,000 protein encoding genes in the genome). It is also accepted that Bonferroni, although mathematically right would be very penalizing for biological data, therefore we opted for techniques based on permutation processes. Our multi-test correction procedure brings together the genotypes of all rare variants in all tested genes as columns into a single table. For each gene a number of markers equal to that of the gene was randomly extracted from this table and its association test calculated. By repeating the procedure for N times an empirical corrected *P*-value was calculated for each tested gene. It can be argued that when randomization is done, LD relations are abrogated affecting the empirical *P*-values computation in random tests, but this problem did not affect our multi-testing correction procedure since we use a working set of independent markers (*r*^2^ < 0.1) (15).

### Enrichment in OMIM Annotations in the “Result-List” of Genes Associated to SLE by Rare Variation

Taking into account that pleiotropic effects on human complex traits was widespread (23), it would be expected that in a list of genes significantly enriched in rare variation there would also be an enrichment of diseases caused by mutations. To test this, we used three data sets. First, we downloaded the OMIM database (http://www.omim.org/downloads/; updated: March 23, 2015), and employed it to build a ‘gene-disease’ table with its 20,707 records (“gene-disease” pairs); second, the list of all GWAS imputed protein-coding genes in our final dataset; and thirdly, the list of *N* genes resulting as candidates to be SLE-associated from our rare variants association analysis. Then if our “result-list” of *N* associated genes provided annotations for *X* OMIM diseases, the procedure for testing enrichment in OMIM annotations was to randomly select a set of *N* genes from the list of GWAS imputed protein-coding genes, and count how many of them appeared on the OMIM “gene-disease” table. This procedure was repeated 1,000 times. The average number of OMIM disease and its standard deviation was calculated and then a Z-score test was performed providing the statistical significance of this enrichment.

## Results

### Imputation

**Figure 1:**
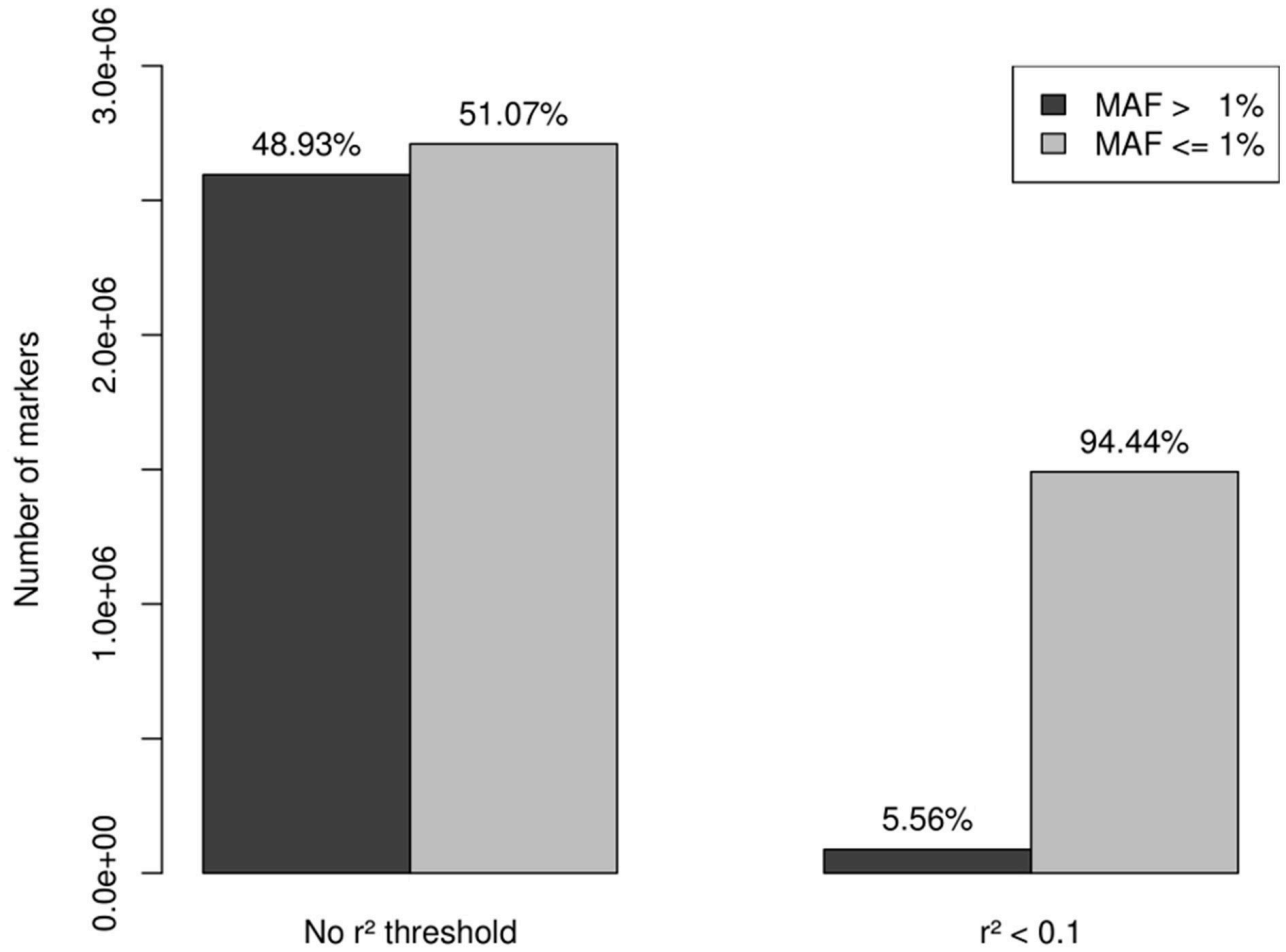
After QC filtering, imputation provided a set of 5,305,811 markers: 2,595,206 with MAF > 1% (48.93%) and 2,709,605 with MAF < 1% (51.07%). A set of 1,549,436 independent markers was obtained by applying a threshold of *r*^2^ < 0.1. Rare variation was less affected by the linkage disequilibrium than common variation, which resulted in 87,853 variants with MAF > 1% (5.56%) and 1,491,583 variants with MAF < 1% (94.44%). This last set of 1,491,583 independent rare-variants constituted our working set.

A total of 13,956 genes passed the QC filter of the imputation process, summing a set of 5,305,811 markers, 2,595,206 variants with MAF > 1% (48.93%), and 2,709,605 variants (mutations) with MAF < 1% (51.07%). A set of 1,549,436 independent markers was obtained by applying a threshold of *r*^2^ < 0.1. As expected, rare variation was much less affected by the linkage disequilibrium than common variation, which resulted in 87,853 variants with MAF > 1% (5.56%) and 1,491,583 variants with MAF < 1% (94.44%) (Figure 1).

**Figure 2:**
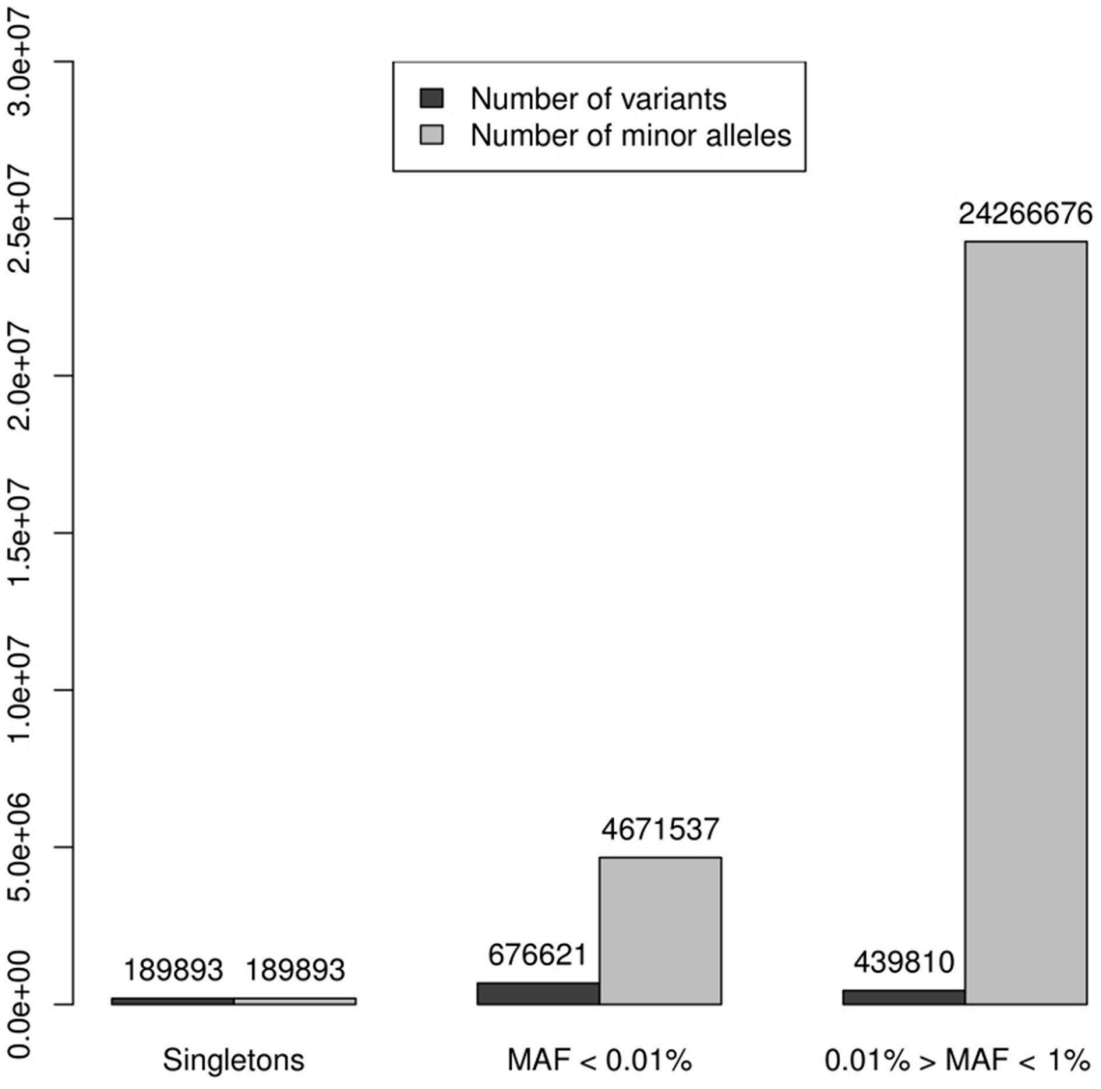
Numbers of variants were represented by dark columns: in the set of 1,306,324 independent (*r*^2^ < 0.1) polymorphic rare variants (MAF < 0.1), 189,893 (14.5%) were singletons (in the 8,277 individuals sample this means a MAF = 0.006%), 676,621 (51.8%) were classified as “very rare-variants” (0.006% < MAF < 0.1%), and 439,810 (33.7%) considered as a rare variation in a “strict sense” (0.1% < MAF ≤ 1%); these numbers of variants were represented by dark columns. Lighter columns represented the sums of alleles of minor frequency in each of the 3 categories of rare variation, these 3 categories sum up 29,128,106 minor alleles: the 189,893 singletons represented only 0.65% of minor alleles; the 676,621 the markers, which were very rare variation, add up to 4,671,537 minor frequency alleles, it was the 16.04%; while the remaining markers, sum up to 24,266,676 of minor frequency alleles representing the 83.33%.

A working set of 1,306,324 independent (*r*^2^ < 0.1) rare variants (MAF < 0.1) was used to test genes for case-control association to the SLE phenotype (185,219 were non-polymorphic). The reliability of these tests depends on the accuracy of the imputation. When analyzing reliability of imputation in rare variants 3 intervals are usually differentiated: “singletons,” “very rare” variation and “rare” variation (4, 24). It is known that as the variant MAF decreases, so does the accuracy of imputation, improving with the size of the reference panel, and singletons, meaning that the minor allele is observed only in one chromosome, are not reliably imputed under any conditions. In our data set, 189,893 (14.5%) variants were singletons (in the 8,277 individuals sample this means a MAF = 0.006%) and if an additional threshold of MAF < 0.1% to distinguish rare variant from a category of “very” rare variant was applied, then 676,621 (51.8%) variants were classified as very rare-variants, while 439,810 (33.7%) had MAF between 0.1 and 1%. These results suggested that imputed genotype data used in the association analysis could be unreliable because of the predominance of markers belonging to the categories of very rare variants and singletons over the most reliable imputation category of rare variation, 0.1 < MAF ≤ 1%. However, in tests based on the combined effect of variants, the main factor is not the number of markers aggregated but more importantly the count of alleles of minor frequency in the sample of analyzed individuals. Thus, in our 8,277 individuals dataset the 3 categories of rare variation sum up 29,128,106 minor alleles. Singletons represented 14.5% of rare variation but only 0.65% of minor alleles. The 51.8% of the markers included in the very rare variation category add up to 4,671,537 minor frequency alleles, that is, 16.04%; while the remaining markers, sum up to 24,266,676 of minor frequency alleles, which represented the 83.33%, resulting in a 5 times greater ratio of “0.1% < MAF ≤ 1%” variation compared to the sum of the other two categories (Table 1, Figure 2). Therefore, despite its lower proportion, the expected effect of rare variation on the aggregate test would be greater than that of the “very rare” variation and singletons providing a higher reliability to the analysis.

**Table 1:** Number of independent rare variants vs. sum of minor alleles.

	**Number of variants**	**Number of minor alleles**
Singletons	189,893 (14.5%)	189,893 (0.64%)
MAF < 0.01%	676,621 (51.8%)	4,671,537 (16.04%)
0.01% > MAF ≤ 1%	439,810 (33.7%)	24,266,676 (83.33%)
	1,306,324 (100%)	29,128,106 (100%)

*Rare variation was classified into 3 categories by their minor allele frequencies (MAFs): (1) singletons that in the 8,277 individuals sample means a MAF = 0.006%, (2) “very rare-variants,” 0.006% < MAF < 0.1%, (3) and rare variation in a “strict sense,” 0.1% < MAF ≤ 1%*.

**Figure 3:**
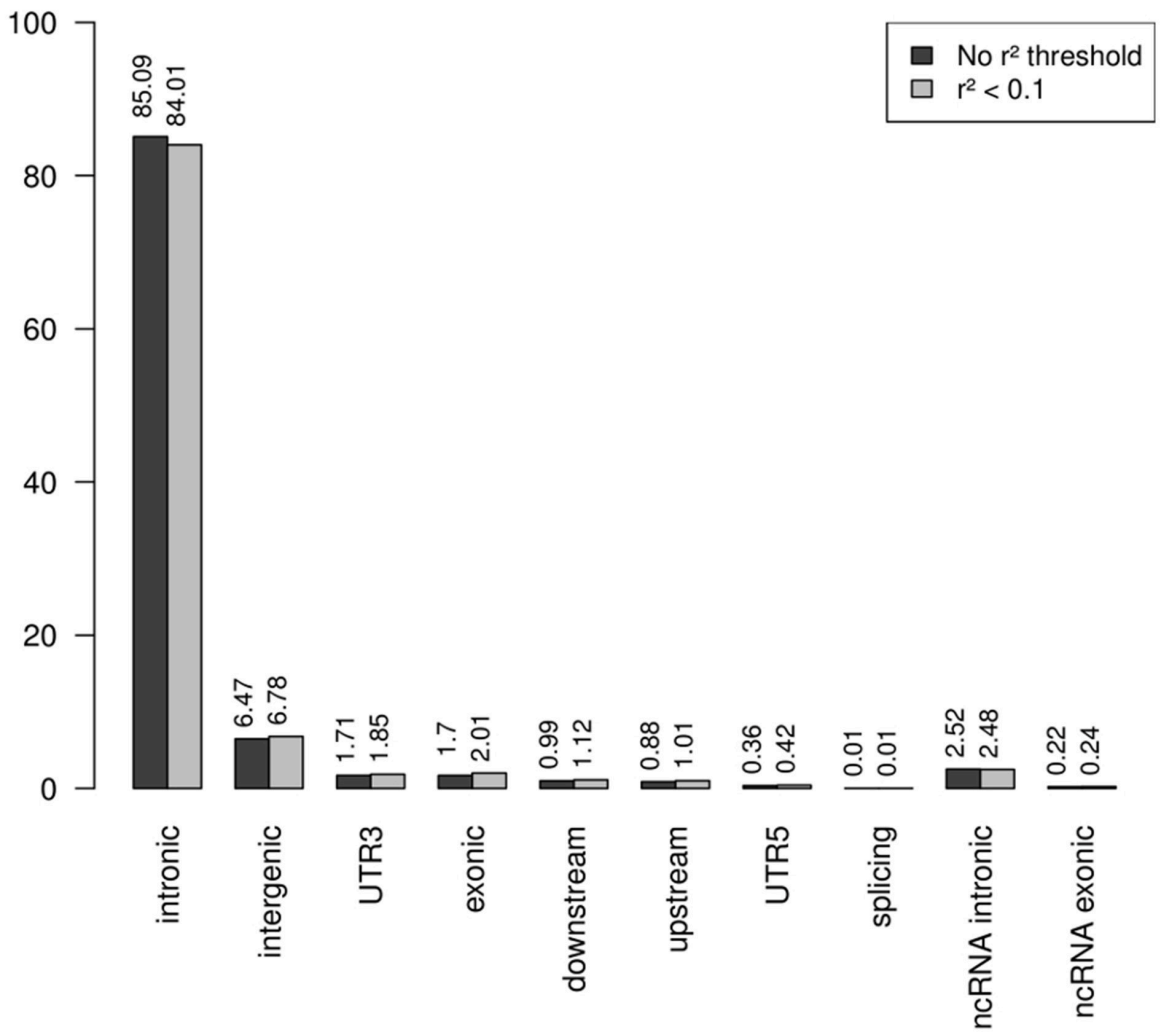
Functional annotation of rare variation. Note that the percentages of the different functional categories in rare variation with and without *r*^2^ filtering was similar, being the intronic the most abundant category, 85% of the total.

**Figure 4:**
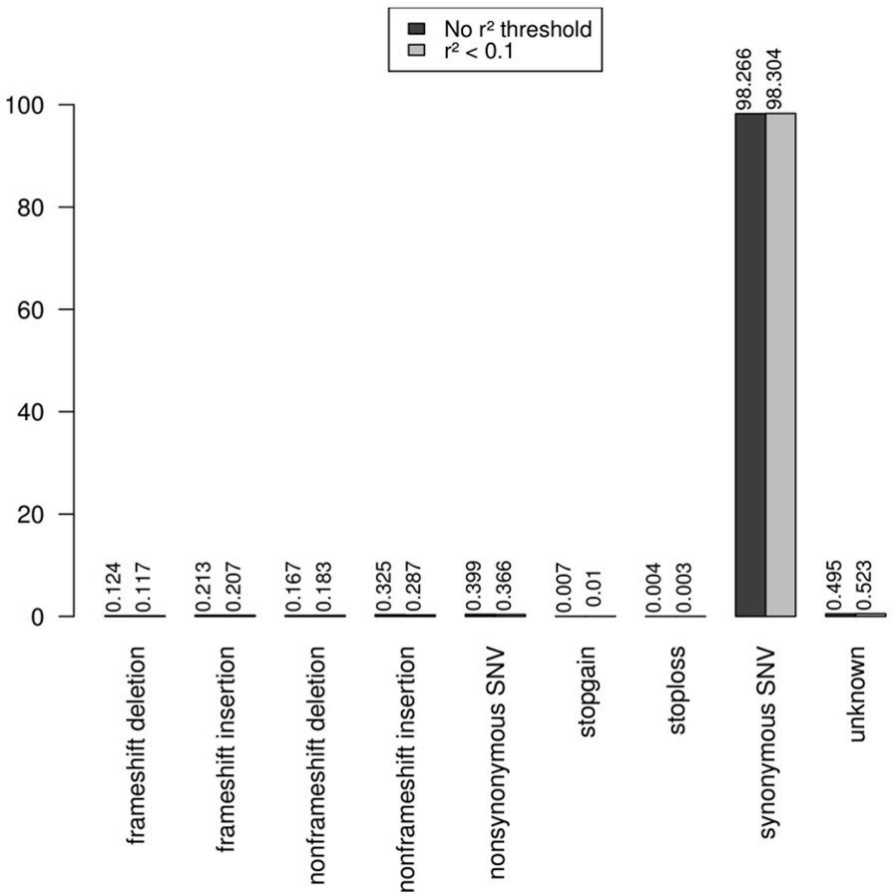
Functional annotation of exonic rare variation. The exonic rare variants represented only 2%, and more than 98% of these exonic mutations were synonymous.

Note that the proportions of the different functional categories in the rare variation (MAF < 1% with and without *r*^2^ filtering was similar (Table 2, Figure 3), being the intronic the most abundant category, 85% of the total, while the exonic rare variants represented only 2%, of which more than 98% were synonymous (Figure 4).

**Table 2:** Functional annotation of rare variation with and without *r*^2^ filtering.

	**No *r*^**2**^ threshold**	***r^2^* < 0.1**
Intronic	2,305,643 (85.09%)	1,253,088 (84.011%)
Intergenic	175,180 (6.465%)	101,187 (6.784%)
UTR3	46,322 (1.71%)	27,637 (1.853%)
Exonic	46,133 (1.70%)	30,053 (2.015%)
Downstream	26,690 (0.985%)	16,688 (1.119%)
Upstream	23,791 (0.878%)	15,095 (1.012%)
UTR5	9,872 (0.364%)	6,215 (0.417%)
Splicing	231 (0.009%)	171 (0.012%)
ncRNA intronic	68,381 (2.524%)	36,967 (2.478%)
ncRNA exonic	6,066 (0.224%)	3,600 (0.241%)

### Rare-Variant Association Gene-Centered Analysis, and OMIM Annotation Enrichment

Under these conditions a set of 281 genes showed SKAT test with Genomic Control and multi-testing corrected *P* < 0.05 (Supplemental Table 1). Noted that 441 genes also presented Genomic Control and multi-testing corrected significant tests for enrichment in rare variation (Supplemental Table 2). When the OMIM annotation enrichment analysis were executed, the list of SKAT associated genes was significantly enriched with 119 OMIM diseases (Supplemental Table 3) instead of the 81 expected at random, which gave a value of *P* = 3E-03. Of these 281 genes, 139 were enriched in mutations in cases vs. controls and the remaining 142 were depleted. Note that the list of 139 genes enriched in mutations had 80 OMIM diseases annotations when expected was just 40, which gave a *P*-value of 6E-05. Remark that the 140 depleted genes showed no significant enrichment in OMIM diseases annotations (39 vs. 41 expected, *P* = 0.59).

As best candidates for SLE association by rare variation, we selected the set of 98 genes which simultaneously showed Genomic Control and multi-testing corrected *P* < 0.05 in both SKAT test and case-control burden test, with the purpose of reducing the proportion of possible spurious associations. These are shown in Table 3. Some of these are discussed as excellent candidates for the identification of individuals with particular clinical phenotypes that may be directly targeted for sequencing. ANNOVAR annotation of the independent mutations mapped on these 98 genes are shown in Supplemental Table 5.

**Table 3:** Best gene candidates for SLE association through rare variation in European ancestry population.

**Gene**	**Description**	**NMUT**	**nMAF.aff**	**nMAF.ctr**	**OR**	**CI._**95lo**_**	**CI_**95up**_**	**P_**burden.test.corr**_**	**PSKAT_**corr**_**
ZEB1	Zinc finger E-box binding homeobox 1	74	1077	886	2.03	1.35	3.05	<1.00E-03	<1.00E-03
PRKAG3	Protein kinase, AMP-activated, gamma 3 non-catalytic subunit	4	53	95	0.55	0.39	0.78	<1.00E-03	<1.00E-03
COQ10B	Coenzyme Q10 homolog B (S. cerevisiae)	3	11	38	0.25	0.13	0.5	<1.00E-03	<1.00E-03
MAD2L2	MAD2 mitotic arrest deficient-like 2 (yeast)	19	417	517	0.59	0.45	0.77	<1.00E-03	3.60E-03
TMEM69	Transmembrane protein 69	5	18	42	0.32	0.19	0.54	<1.00E-03	4.40E-03
KRTAP9-2	Keratin associated protein 9-2	3	4	14	0.16	0.05	0.5	<1.00E-03	6.20E-03
SERINC4	Serine incorporator 4	4	27	4	5.98	2.23	16.03	<1.00E-03	6.40E-03
CCR3	Chemokine (C-C motif) receptor 3	33	750	431	3.74	1.74	8.02	<1.00E-03	8.40E-03
CYP26B1	Cytochrome P450, family 26, subfamily B, polypeptide 1	20	170	93	1.7	1.31	2.21	<1.00E-03	1.01E-02
TMEM106B	Transmembrane protein 106B	24	103	244	0.56	0.38	0.82	<1.00E-03	1.77E-02
POU3F3	POU class 3 homeobox 3	2	66	33	3.47	1.46	8.22	<1.00E-03	2.41E-02
TIGD7	Tigger transposable element derived 7	14	109	55	3.04	1.58	5.86	1.80E-03	1.39E-02
KLF1	Kruppel-like factor 1 (erythroid)	3	90	48	3.01	1.46	6.2	1.80E-03	2.29E-02
PSMB8	Proteasome (prosome, macropain) subunit, beta type, 8 (large multifunctional peptidase 7)	30	439	490	0.64	0.48	0.84	1.80E-03	2.46E-02
MAPK15	Mitogen-activated protein kinase 15	6	55	78	0.57	0.4	0.82	1.80E-03	2.67E-02
AMACR	Alpha-methylacyl-CoA racemase	20	347	261	1.64	1.17	2.29	1.80E-03	2.77E-02
UBE3A	Ubiquitin protein ligase E3A	73	1164	1340	0.53	0.35	0.82	1.80E-03	3.93E-02
TRIM16L	Tripartite motif containing 16-like	13	179	227	0.52	0.34	0.78	3.40E-03	<1.00E-03
CTSK	Cathepsin K	3	23	41	0.26	0.1	0.68	3.40E-03	6.20E-03
HYPK	Huntingtin interacting protein K	3	33	6	13.76	2.45	77.3	3.40E-03	8.30E-03
C4A	Complement component 4A (Rodgers blood group)	2	17	33	0.43	0.23	0.79	3.40E-03	1.40E-02
WNT10A	Wingless-type MMTV integration site family, member 10A	16	153	215	0.52	0.34	0.81	3.40E-03	1.57E-02
OR5B12	Olfactory receptor, family 5, subfamily B, member 12	3	98	141	0.44	0.26	0.75	3.40E-03	1.78E-02
TAGLN3	Transgelin 3	27	225	275	0.58	0.4	0.84	3.40E-03	2.62E-02
PALM	Paralemmin	65	653	740	0.52	0.34	0.79	3.40E-03	2.84E-02
SPACA1	Sperm acrosome associated 1	25	405	279	2.02	1.3	3.12	4.80E-03	6.10E-03
POPDC2	Popeye domain containing 2	36	444	309	2.47	1.39	4.4	4.80E-03	6.30E-03
ALG11	Asparagine-linked glycosylation 11, alpha-1,2-mannosyltransferase homolog (yeast)	10	60	91	0.59	0.42	0.83	4.80E-03	7.90E-03
SF3B4	Splicing factor 3b, subunit 4, 49kDa	3	58	88	0.39	0.2	0.78	4.80E-03	1.78E-02
DUSP7	Dual specificity phosphatase 7	4	32	12	8.12	2.03	32.54	4.80E-03	3.11E-02
MRGPRX4	MAS-related GPR, member X4	9	74	108	0.62	0.46	0.85	6.30E-03	7.50E-03
AQP8	Aquaporin 8	9	141	83	2.5	1.42	4.41	6.30E-03	1.15E-02
PI3	Peptidase inhibitor 3, skin-derived	2	20	4	3.85	1.29	11.5	6.30E-03	1.58E-02
FRS3	Fibroblast growth factor receptor substrate 3	28	384	447	0.66	0.49	0.87	6.30E-03	2.78E-02
MICB	MHC class I polypeptide-related sequence B	47	1110	1166	0.62	0.45	0.84	6.30E-03	2.97E-02
GPBP1L1	GC-rich promoter binding protein 1-like 1	10	172	193	0.57	0.38	0.86	7.70E-03	<1.00E-03
DOLK	Dolichol kinase	1	15	3	5.66	1.59	20.2	7.70E-03	9.90E-03
CSTA	Cystatin A (stefin A)	23	266	200	2.26	1.28	3.97	7.70E-03	1.59E-02
KLHL31	Kelch-like 31 (Drosophila)	39	465	518	0.51	0.32	0.81	7.70E-03	1.61E-02
GPR26	G protein-coupled receptor 26	58	934	734	332	1.25	2.94	7.70E-03	1.65E-02
PRDM1	PR domain containing 1, with ZNF domain	26	278	332	0.62	0.44	0.86	7.70E-03	2.45E-02
COX17	COX17 cytochrome c oxidase assembly homolog (S. cerevisiae)	32	398	280	2.13	1.31	3.46	7.70E-03	3.26E-02
SAMSN1	SAM domain, SH3 domain and nuclear localization signals 1	129	1977	1622	1.74	1.21	2.5	9.10E-03	1.20E-02
ST8SIA2	ST8 alpha-N-acetyl-neuraminide alpha-2,8-sialyltransferase 2	153	1583	1653	0.51	0.32	0.81	9.10E-03	1.75E-02
MAS1L	MAS1 oncogene-like	12	141	171	0.52	0.32	0.83	9.10E-03	3.38E-02
TBCB	Tubulin folding cofactor B	21	236	174	1.66	1.14	2.42	1.05E-02	<1.00E-03
FGR	Gardner-Rasheed feline sarcoma viral (v-fgr) oncogene homolog	6	154	93	1.97	1.17	3.3	1.05E-02	2.67E-02
WNK4	WNK lysine deficient protein kinase 4	5	161	116	2.06	1.25	3.41	1.05E-02	4.41E-02
MGAT5	Mannosyl (alpha-1,6-)-glycoprotein beta-1,6-N-acetyl-glucosaminyltransferase	426	3961	3984	0.51	0.33	0.79	1.18E-02	2.64E-02
FEZF1	FEZ family zinc finger 1	3	49	74	0.37	0.18	0.79	1.18E-02	4.02E-02
ZNF461	Zinc finger protein 461	6	55	80	0.37	0.18	0.77	1.32E-02	2.34E-02
RAB25	RAB25, member RAS oncogene family	8	82	110	0.45	0.25	0.81	1.32E-02	3.94E-02
APLNR	Apelin receptor	5	65	44	2.67	1.21	5.87	1.45E-02	1.07E-02
DUS1L	Dihydrouridine synthase 1-like (S. cerevisiae)	15	58	89	0.38	0.19	0.75	1.45E-02	2.60E-02
ASAH2	N-acylsphingosine amidohydrolase (non-lysosomal ceramidase) 2	16	256	177	2.34	1.3	4.24	1.45E-02	2.76E-02
MRPS18B	Mitochondrial ribosomal protein S18B	23	554	584	0.61	0.42	0.88	1.58E-02	1.76E-02
IL18RAP	Interleukin 18 receptor accessory protein	22	278	213	1.62	1.12	2.34	1.58E-02	2.77E-02
BNIP2	BCL2/adenovirus E1B 19kDa interacting protein 2	21	196	246	0.59	0.4	0.87	1.58E-02	2.93E-02
HAX1	HCLS1 associated protein X-1	2	47	17	2.04	1.16	3.61	1.58E-02	3.08E-02
ITFG3	Integrin alpha FG-GAP repeat containing 3	72	993	1076	0.5	0.3	0.8	1.58E-02	3.63E-02
ZNF99	Zinc finger protein 99	8	18	32	0.24	0.08	0.74	1.71E-02	1.51E-02
ZIK1	zinc finger protein interacting with K protein 1 homolog (mouse)	15	173	116	1.9	1.18	3.06	1.71E-02	2.43E-02
PDZK1	PDZ domain containing 1	12	324	376	0.67	0.48	0.92	1.84E-02	2.43E-02
KRCC1	Lysine-rich coiled-coil 1	28	272	203	1.64	1.12	2.41	1.84E-02	4.45E-02
MAP1LC3C	Microtubule-associated protein 1 light chain 3 gamma	10	161	206	0.58	0.38	0.89	1.97E-02	2.24E-02
LRP4	Low density lipoprotein receptor-related protein 4	1	12	2	5.28	1.16	23.99	1.97E-02	2.59E-02
OR52K2	Olfactory receptor, family 52, subfamily K, member 2	14	158	196	0.57	0.36	0.88	1.97E-02	4.29E-02
GML	Glycosylphosphatidylinositol anchored molecule like protein	66	427	496	0.45	0.25	0.8	2.10E-02	1.30E-02
ARL4A	ADP-ribosylation factor-like 4A	19	163	97	1.96	1.17	3.28	2.10E-02	3.85E-02
ANKRD39	Ankyrin repeat domain 39	3	36	55	0.58	0.38	0.9	2.22E-02	1.95E-02
HLA-DRB1	Major histocompatibility complex, class II, DR beta 1	6	52	71	0.4	0.19	0.83	2.22E-02	2.51E-02
PNO1	Partner of NOB1 homolog (S. cerevisiae)	8	79	44	2.49	1.17	5.31	2.22E-02	3.94E-02
LCE2D	Late cornified envelope 2D	3	45	49	0.38	0.17	0.86	2.22E-02	4.75E-02
ANAPC11	Anaphase promoting complex subunit 11	1	18	33	0.52	0.29	0.94	2.35E-02	4.86E-02
OR7A17	Olfactory receptor, family 7, subfamily A, member 17	7	109	73	2.15	1.16	4.01	2.47E-02	3.93E-02
PTGDS	Prostaglandin D2 synthase 21kDa (brain)	17	166	124	1.76	1.09	2.86	2.60E-02	1.01E-02
CCR1	Chemokine (C-C motif) receptor 1	8	149	89	1.81	1.09	3	2.72E-02	7.30E-03
CCDC12	Coiled-coil domain containing 12	29	380	272	1.98	1.13	3.47	2.97E-02	1.95E-02
REEP4	Receptor accessory protein 4	10	115	83	2.03	1.13	3.65	3.09E-02	3.22E-02
ZNF513	Zinc finger protein 513	3	18	29	0.51	0.28	0.94	3.09E-02	3.56E-02
SPATA8	Spermatogenesis associated 8	19	145	100	1.87	1.1	3.16	3.22E-02	3.60E-03
TAF15	TAF15 RNA polymerase II, TATA box binding protein (TBP)-associated factor, 68kDa	49	439	346	1.89	1.12	3.19	3.22E-02	3.13E-02
POLR2I	Polymerase (RNA) II (DNA directed) polypeptide I, 14.5kDa	11	142	96	1.7	1.07	2.71	3.22E-02	3.84E-02
KCTD5	Potassium channel tetramerisation domain containing 5	24	217	250	0.54	0.33	0.89	3.34E-02	1.59E-02
NKX2-5	NK2 homeobox 5	11	109	74	2.06	1.11	3.79	3.46E-02	3.23E-02
SCARA3	Scavenger receptor class A, member 3	69	593	469	1.68	1.09	2.59	3.46E-02	4.51E-02
EFNA4	Ephrin-A4	7	37	51	0.39	0.16	0.93	3.70E-02	2.52E-02
NRM	Nurim (nuclear envelope membrane protein)	11	229	233	0.65	0.44	0.95	3.82E-02	2.26E-02
TMEM55B	Transmembrane protein 55B	8	72	45	1.57	1.06	2.31	3.94E-02	9.30E-03
MPL	Myeloproliferative leukemia virus oncogene	15	83	44	2.48	1.16	5.31	4.06E-02	1.93E-02
PTGS2	Prostaglandin-endoperoxide synthase 2 (prostaglandin G/H synthase and cyclooxygenase)	6	79	109	0.29	0.1	0.86	4.18E-02	3.61E-02
NT5DC1	5'-nucleotidase domain containing 1	87	736	558	2.43	1.09	5.41	4.18E-02	4.54E-02
MAFG	v-maf musculoaponeurotic fibrosarcoma oncogene homolog G (avian)	7	68	90	0.69	0.5	0.97	4.54E-02	1.11E-02
C5orf28	Chromosome 5 open reading frame 28	10	72	90	0.52	0.28	0.99	4.54E-02	3.22E-02
C8orf58	Chromosome 8 open reading frame 58	19	347	413	0.6	0.38	0.95	4.66E-02	3.85E-02
SNX5	Sorting nexin 5	63	490	374	1.99	1.08	3.64	4.77E-02	2.51E-02
PTER	Phosphotriesterase related	98	846	716	2.11	1.06	4.2	4.89E-02	5.50E-03
ZNF708	Zinc finger protein 708	8	68	88	0.54	0.31	0.96	4.89E-02	1.69E-02

*We selected the set of 98 genes which simultaneously showed Genomic Control and multi-testing corrected P-values < 0.05 in both SKAT test and Case-Control burden test (Case-Control burden test helps to interpret the effect that the rare variation on each associated gene had on the phenotype, which is measured by the overall OR value and its 95% confidence interval)*.

*NMUT, number of mutations on tested gene; nMAF.aff, sum of minor frequency alleles in nMUT mutations in cases (4,212 cases); nMAF.ctr, sum of minor frequency alleles in nMUT mutations in controls (4,065 controls); OR, case-control burden test Odds Ratio; CI._95lo_, case-control burden test 95% Confidence Interval minor value; CI._95up_: case-control burden test 95% Confidence Interval major value; P_burden.test.corr_, case-control burden test corrected P-value; and P_SKAT.corr_, SKAT corrected P-value*.

## Discussion

We have described a strategy to identify the association of rare variation with a complex disease based on densely genotyped data and stringent imputation. Our study provides a first list of genes potentially involved in SLE through rare mutations that may have an impact on the clinical presentation of the disease.

Although it could seem difficult to justify the role of non-coding rare gene variation as causal, there are numerous examples that support it. Efforts to identify risk alleles usually are focused on exploring coding mutation by exome sequencing, noting Pullabhatla et al. (25), as a recent example in SLE, but analogous works for non-coding variants are scant. As examples supporting the causative role of rare non-coding gene variation in these complex phenotypes, it has been recently reported that non-noding mutation affected plasma lipid traits in a founder population (26), or in a more generalized perspective, it has been demonstrated that rare variants contribute to large gene expression changes across tissues and provide an integrative method for interpretation of rare variants effects (27).

It is important to point out that a significant test for aggregated case-control burden test would indicate that in the set of individuals forming the sample, the overall effect of the rare variation on the gene goes in the same direction being either of risk or protective. This feature of case-control burden analysis helps to interpret the effect of rare variation on the phenotype, which is measured by the overall OR values, OR > 1 or OR < 1, risk or protection, respectively. Mutations associated with diseases were usually considered to be detrimental to health, increasing the risk of disease. However, there are a growing number of reported mutations shown to be protective, lowering the risk of certain diseases and conditions (28–31). In this context we can explain why the gene STAT4 associated to SLE by common variation (32–45) and our 20th SKAT best-hit, failed the burden test (Supplemental Tables 1, 4). There were two sets of rare variation mapping on the gene with opposite join effects and therefore reducing the power of the overall burden test. The same rationale could be applied to the other SLE associated genes by common variation also detected as targets by rare variation with SKAT but not by burden test in our study (Supplemental Tables 1, 4): GTF2IRD1 (39, 42), DAB2 (41), NOTCH4 (37), CLEC16A (32, 42, 44), TNFSF4 (32, 40–46), and C2 (36). The same can be argued for DOCK8 (OR_burden.test_ = 2.33, P _SKAT.corr_ = 0.03 and P_burden.test.corr_ = 0.204) the cause of Hyper-IgE recurrent infection syndrome (HIES) autosomal recessive by homozygous or compound heterozygous mutation (OMIM #243700). Note that it has been reported a case of DOCK8 deficiency caused by a truncating mutation, associated with SLE (47).

Focusing on the best-hits which meet the criteria of simultaneous significant association in both rare variation tests, SKAT and burden test, we found some genes described as associated with SLE by common variation such as TMEM55B (46), SPATA8 (36), PRDM1 (32, 40, 42, 44, 48), and HLA-DRB1 (36, 40, 42, 43, 45) (Table 3). Note IRF7 also associated to SLE through common variants (32, 38, 44) had association values through rare variation close to statistical significance (P_SKAT.corr_ = 0.082 and P_burden.test.corr_ = 0.036) (Supplemental Table 4).

In this kind of studies it is usual to obtain lists of genes with a difficult functional justification. However, in this case we can relate many of the best hits in Table 3 directly to immunological functions or with effects on other organs or tissues affected by SLE, such as skin, central nervous system or blood. For example our list contained C4A, P_SKAT.corr_ = 0.014 and P_burden.test.corr_ = 0.0034, OR_burden.test_ = 0.43 and CI_95%.burden.test_ = (0.23, 0.79), which was early related to SLE through mutation [OMIM: 152700; (49)]. The zinc finger E-box binding homeobox 1 gene, ZEB1, P _SKAT.corr_ < 1.00E-03 and P_burden.test.corr_ < 1.00E-03, OR_enrich_ = 2.03 and CI _95%.enrich_ = (1.35, 3.05), acts as a transcriptional repressor inhibiting interleukin-2 (IL-2) gene expression. Note that IL-2 plays a critical role in immune tolerance, and insufficient IL-2 production upon stimulation has been recognized in SLE pathogenesis, particularly it has been described a new epigenetic pathway in the control of IL-2 production in SLE whereby low levels of miR-200a-3p accumulate the binding of the ZEB1-CtBP2 complex to the IL-2 promoter and suppresses IL-2 production (50). The role of CCR3 [P _SKAT.corr_ = 0.00838 and P_burden.test.corr_ < 1.00E-03, OR_burden.test_ = 3.74 and CI _95%.burden.test_ = (1.74, 8.02)] in inflammation is widely known (OMIM ^*^601268).

The protein encoded by CYP26B1, P _SKAT.corr_ = 0.01 and P_burden.test.corr_ < 1.00E-03, OR_burden.test_ = 1.7 and CI _95%.burden.test_ = (1.31, 2.21), functions as a critical regulator of all-trans retinoic acid levels by the specific inactivation of all-trans retinoic acid to hydroxylated forms. Mast cells (MCs) are known to be regulators of inflammation. It has been reported that the ATP-P2X7 pathway induces MCs activation and consequently exacerbates inflammation. P2X7 expression on MCs was reduced by fibroblasts in the skin. Cyp26b1 was highly expressed in skin fibroblasts. Cyp26b1 inhibition resulted in upregulation of P2X7 on MCs and the presence of excessive amounts of retinoic acid correlated with the increased expression of P2X7 on skin MCs and consequent P2X7- and MC-dependent dermatitis (so-called retinoid dermatitis) (51).

The protein encoded by TIGD7 belongs to the “tigger subfamily of the pogo superfamily of DNA-mediated transposons” in humans, P _SKAT.corr_ = 0.0139 and P_burden.test.corr_ = 0.00179, OR_burden.test_ = 3.04 and CI_95%.burden.test_ = (1.58, 5.86). The exact function of this gene is not known, but it is very similar to CENPB considered a major centromere auto-antigen recognized by sera from patients with anti-cetromere-antibodies (ACA), which occur in some autoimmune diseases, frequently in limited systemic scleroderma and occasionally in its diffuse form (52, 53).

AQP8, P _SKAT.corr_ = 0.0115 and P_burden.test.corr_ = 0.0063, OR_burden.test_ = 2.5 and CI _95%.burden.test_ = (1.42, 4.41). It has been reported that efficient induction of B cell activation and differentiation requires H_2_O_2_ fluxes across the plasma membrane for signal amplification. NADPH-oxidase 2 is the main source of H_2_O_2_ and AQP8 is the transport facilitator across the plasma membrane. AQP8 silencing inducible B lymphoma cells responded poorly to TLR and BCR stimulation. Conversely a silencing-resistant AQP8 rescued responsiveness (54). In addition AQP8 was the major antibody target on human salivary glands in patients with primary Sjögren's syndrome (55).

It was known that SAMSN1 (=HACS1), P _SKAT.corr_ = 0.012 and P_burden.test.corr_ = 0.0091, OR_burden.test_ = 1.74 and CI_95%.burden.test_ = (1.21, 2.5), is up-regulated by B cell activation signals and it participates in B cell activation and differentiation (56).

MICB, P _SKAT.corr_ = 0.0297 and P_burden.test.corr_ = 0.0062, OR_burden.test_ = 0.62 and CI _95%.burden.test_ = (0.45, 0.84), acts as a stress-induced self-antigen that is recognized by gamma delta T cells. MICB might play a role in both SLE and cutaneous LE (CLE) in european population (57). In addition MICB has been associated with susceptibility to SLE in Han Chinese Population (58, 59).

CTSK, P _SKAT.corr_ = 0.0062 and P_burden.test.corr_ = 0.0034, OR_burden.test_ = 0.28 and CI_95%.burden.test_ = (0.1, 0.68), is highly expressed by rheumatoid synovial fibroblasts (RSF) that are activated by toll-like receptor signaling pathways in rheumatoid arthritis and is known to play a key role in the degradation of type Iand type II collagen. Thus, cathepsin K is implicated in the degradation of bone and cartilage in RA (60). In addition it has been suggested that CTSK is involved in development of psoriasis-like skin lesions through TLR-dependent Th17 activation (61).

Autoinflammation, lipodystrophy, and dermatosis syndrome (ALDD) can be caused by homozygous mutations in the PSMB8 gene (OMIM: # 256040), P _SKAT.corr_ = 0.0246 and P_burden.test.corr_ = 0.00179, OR_burden.test_ = 0.64 and CI _95%.burden.test_ = (0.48, 0.84). This autosomal recessive systemic autoinflammatory disorder is characterized by early childhood onset of annular erythematous plaques on the face and extremities with subsequent development of partial lipodystrophy and laboratory evidence of immune dysregulation. More variable features include recurrent fever, severe joint contractures, muscle weakness and atrophy, hepatosplenomegaly, basal ganglia calcifications, and microcytic anemia (62–64). This disorder encompasses Nakajo-Nishimura syndrome (NKJO); joint In contractures, muscular atrophy, microcytic anemia, and panniculitis-induced lipodystrophy (JMP syndrome); and chronic atypical neutrophilic dermatosis with lipodystrophy and elevated temperature syndrome (CANDLE). Furthermore, mutations in PSMB8 and other proteasome unit genes were shown to lead to an increased type I interferon signature (65), a characteristic of SLE.

The roles of TRIM16L, P _SKAT.corr_ = 0.0033 and P_burden.test.corr_ < 1.00E-03, OR_burden.test_ = 0.52 and CI_95%.burden.test_ = (0.34, 0.78), in immune response are unknown, however it has been reported that in fish models TRIM16L exerted negative regulation of the interferon immune response against DNA virus infection (66). The early events that facilitate viral persistence in chronic viral infections have been linked to the activity of the immunoregulatory cytokine IL-10. It has been reported that IL-10 induced the expression of MGAT5, a glycosyltransferase that enhances N-glycan branching on surface glyco- proteins, P _SKAT.corr_ = 0.0264 and P_burden.test.corr_ = 0.0118, OR_burden.test_ = 0.51 and CI _95%.burden.test_ = (0.33, 0.79). Increased N-glycan branching on CD8+ T cells promoted the formation of a galectin 3-mediated membrane lattice, which restricted the interaction of key glycoproteins, ultimately increasing the antigenic threshold required for T cell activation allowing the establishment of chronic infection (67).

The serine incorporator 4, SERINC4, P SKAT.corr = 0.0064 and P_burden.test.corr_ < 1.00E-03, OR_burden.test_ = 5.98 and CI_95%.burden.test_ = (2.23, 16.03), incorporates amino acid serine into membranes and facilitates the synthesis of two serine-derived lipids, phosphatidylserine and sphingolipids (68).

Gene KLF1, P_SKAT.corr_ = 0.00179 and P_burden.test.corr_ = 0.0229, OR_burden.test_ = 3.01 and CI_95%.burden.test_ = (1.46, 6.20), encodes a hematopoietic-specific transcription factor that induces high-level expression of adult beta-globin and other erythroid genes. Heterozygous loss-of-function mutations in this gene result in the dominant In(Lu) blood phenotype (69). Compound heterozygosity for KLF1 mutations is associated with microcytic hypochromic anemia and increased fetal hemoglobin (70). Mutations in KLF1 cause dyserythropoietic anemia congenital type IV (OMIM: 613673).

TMEM106B [P _SKAT.corr_ < 1.00E-03 and P_burden.test.corr_ = 0.0177, OR_burden.test_ = 0.56 and CI_95%.burden.test_ = (0.38, 0.82)] was associated with frontotemporal dementia (71, 72). In addition TMEM106B has been associated with inflammation, neuronal loss, and cognitive deficits, even in the absence of known brain disease, and their impact is highly selective for the frontal cerebral cortex of older individuals (73).

Mutations affecting the gene ALG11 [P_SKAT.corr_ = 0.0079 and P_burden.test.corr_ = 0.0048, OR_burden.test_ = 0.56 and CI_95%.burden.test_ = (0.42, 0.83)] cause congenital disorder of glycosylation 1P (CDG1P) [OMIM: 613661], a multisystem disorder caused by a defect in glycoprotein biosynthesis and characterized by under-glycosylated serum glycoproteins. Congenital disorders of glycosylation result in a wide variety of clinical features, such as defects in the nervous system development, psychomotor retardation, dysmorphic features, hypotonia, coagulation disorders, and immunodeficiency (74, 75).

MAD2L2, P_SKAT.corr_ < 0.001 and P_burden.test.corr_ = 0.0036, OR_burden.test_ = 0.59 and CI_95%.burden.test_ = (0.45, 0.77), controls DNA repair at telomeres and DNA breaks by inhibiting 5' end resection (76). Note that a role for MAPK15 (=ERK8), P _SKAT.corr_ = 0.0018 and P _enrich.corr_ = 0.0267, OR _enrich_ = 0.57 and CI _95%.enrich_ = (0.40,0.77), in the response to, or repair of, DNA single strand breaks has been proposed (77), and it is annotated as “positive regulation of telomerase activity,” biological process (GO:0051973). MRGPRX4 (= MrgX4) [P _SKAT.corr_ = 0.0063 and P_burden.test.corr_ = 0.00748, OR_burden.test_ = 0.62 and CI_95%.burden.test_ = (0.46, 0.85)] is a Mas-related G-protein coupled receptor X (MrgXs). It was described as an oncogene in human colorectal cancers (78), however, it has recently been linked to immunological functions. AG-30/5C is an angiogenic host defense peptide (HDP) that activates various functions of fibroblasts and endothelial cells, including cytokine/chemokine production and wound healing. It has been shown that AG-30/5C enhanced the production of cytokines/chemokines and facilitated keratinocyte migration and proliferation mainly via MrgX3 and MrgX4 receptors constitutively expressed in keratinocytes and up-regulated upon stimulation with TLR ligands. AG-30/5C-induced activation of keratinocytes was controlled by MAPK and NF-κB pathways (79).

In addition other genes associated to human energy metabolism and more specifically in the mitochondrion, as part of the respiratory chain, the best hit associated to SLE by rare variation was COQ10B, P_SKAT.corr_ < 0.001 and P_burden.test.corr_ < 0.001, OR_burden.test_ = 0.25 and CI_95%.burden.test_ = (0.13, 0.50). It encodes coenzyme Q, an essential component of the electron transport chain. The copper metallochaperone COX17, P _SKAT.corr_ = 0.0077 and P_burden.test.corr_ = 0.0326, OR_burden.test_ = 2.13 and CI_95%.burden.test_ = (1.31, 3.46), is essential for the assembly and activation of the cytochrome c oxidase complex (80), the terminal component of the mitochondrial respiratory chain that catalyzes the electron transfer from reduced cytochrome c to oxygen. Null mutations in COX17 elicit a loss of cytochrome oxidase due to the failure of the mutants to complete assembly of the complex [OMIM: ^*^604813]. It has been reported that SLE T-cells have persistently hyperpolarized mitochondria associated with increased mitochondrial mass, high levels of reactive oxygen species (ROS) and low levels of ATP. These hyperpolarized mitochondria resist the depolarization required for activation-induced apoptosis and predispose T cells for necrosis, thus stimulating inflammation in SLE (81, 82). Necrotic cell lysates activate dendriticcells and might account for increased interferon a production and inflammation in lupus patients (83). Additionally, the mitochondrial ATP deficits also reduce the macrophage energy that is needed to clear apoptotic bodies (84). The mitochondrial transmembrane potential is result of the respiratory electron transport chain that drives the flow of electrons from NADH to molecular oxygen by its last enzyme the cytochrome c oxidadase. Note that it has been reported that COX17 is essential for activation of cytochrome C oxidase (80) linking the COX17 function with the SLE phenotype.

## Conclusions

Here we present a set of 98 genes as good candidates for association with SLE by mutation affecting a diversity of functions in different organs and tissues. Considering that complex phenotypes involves the intervention of multiple genes associated by common variation, the same scheme could be expected for genes associated by rare variation. Thus, each gene or set of genes would influence in a small group of affected carriers explaining the clinical heterogeneity or complexity of this pathology. However, it is necessary to remark that these results are preliminary and would need to be confirmed by sequencing in the best candidate carriers in our sample data set.

## Author Contributions

MM-B contributed to the conception and design of the study, organized the database, performed the statistical analysis, wrote the first draft of the manuscript, and participated in the manuscript revision. MA-R provided the raw genotype data sets, contributed to the conception and design of the study, interpretation of results, manuscript revision and wrote the final draft of the manuscript.

### Conflict of Interest Statement

The authors declare that the research was conducted in the absence of any commercial or financial relationships that could be construed as a potential conflict of interest.
