# Supplementary figures and images for: Exploring Impact of Rare Variation in Systemic Lupus Erythematosus by a Genome Wide Imputation Approach
Source: Front Immunol. 2019 Feb 26;10:258. doi: 10.3389/fimmu.2019.00258 (PMC6399402; doi:10.3389/fimmu.2019.00258)

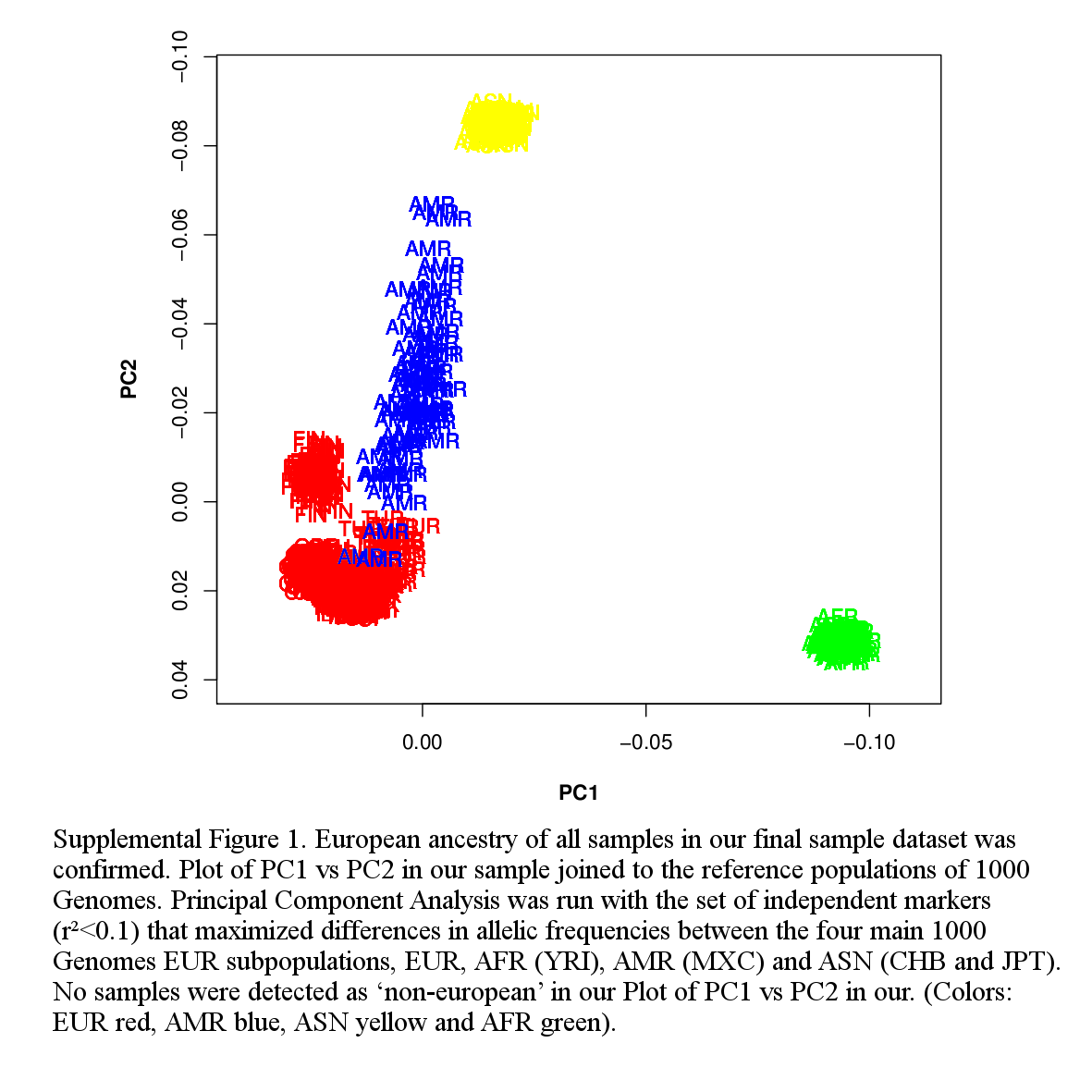

Supplement: Supplementary file 6 [file Image_1.PNG]

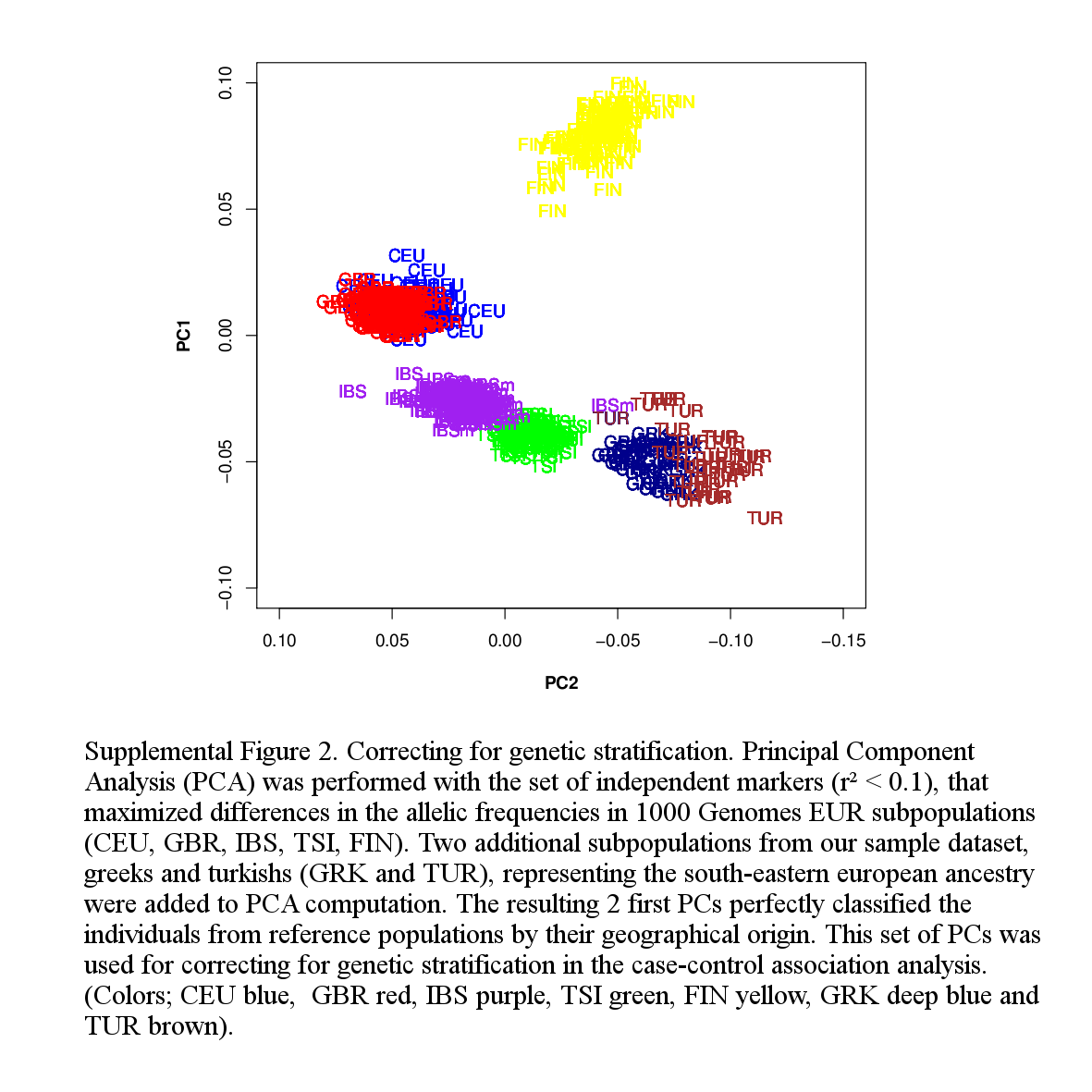

Supplement: Supplementary file 7 [file Image_2.PNG]

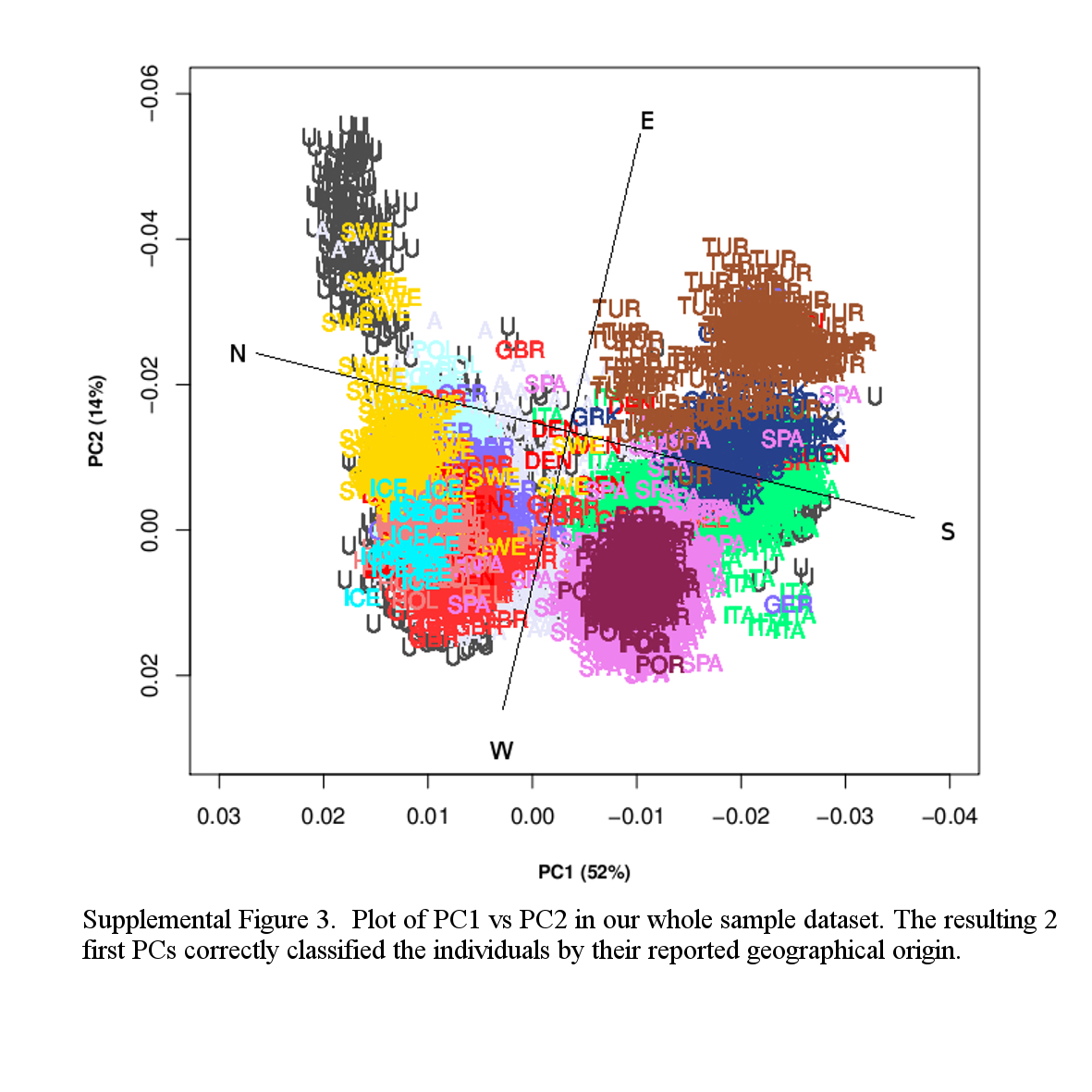

Supplement: Supplementary file 8 [file Image_3.PNG]

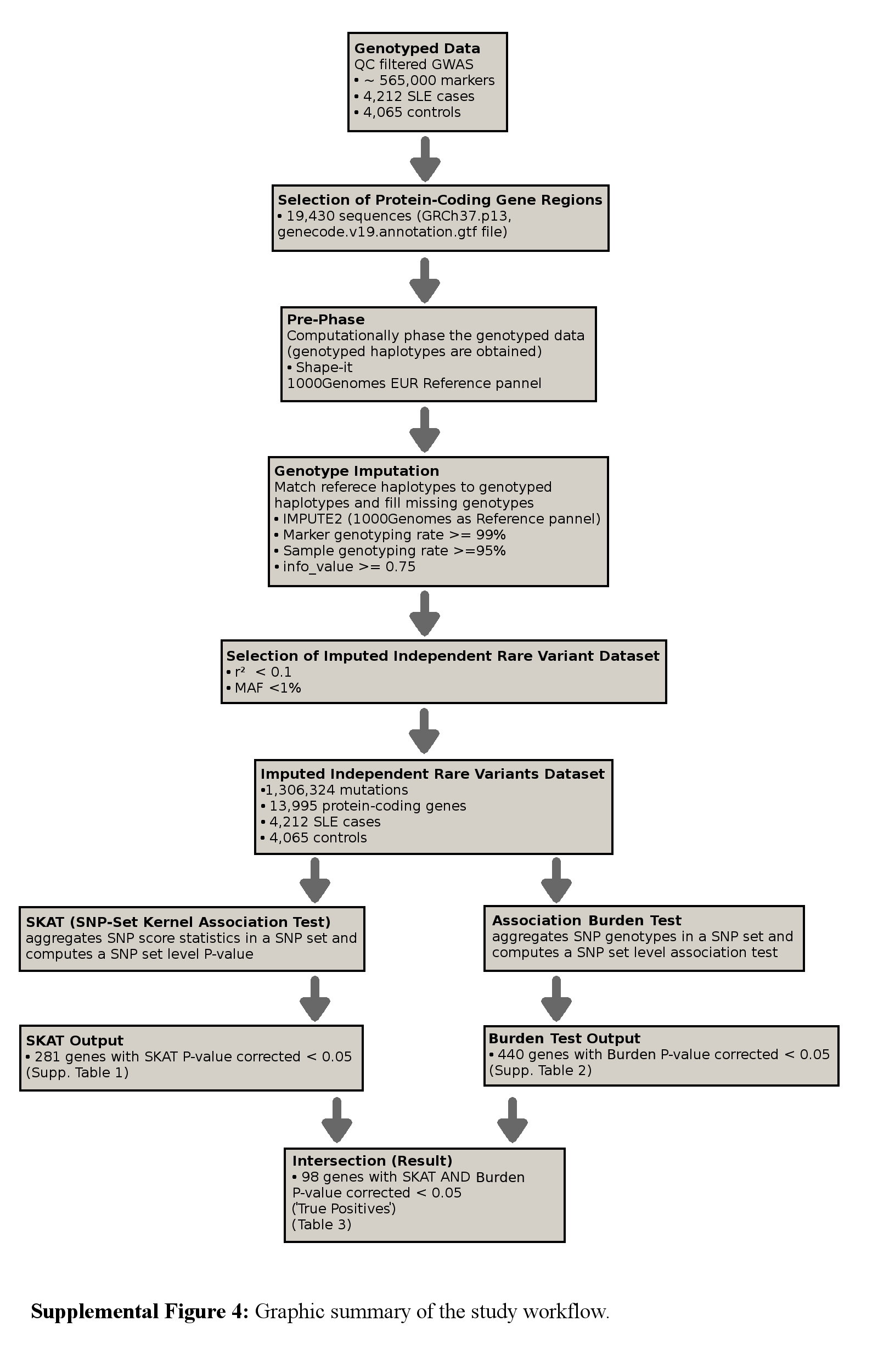

Supplement: Supplementary file 9 [file Image_4.png]
